# Machine Learning-Based Analyses of the Effects of Various Types of Air Pollutants on Hospital Visits by Asthma Patients

**DOI:** 10.3390/toxics10110644

**Published:** 2022-10-27

**Authors:** Soyeon Lee, Hyeeun Ku, Changwan Hyun, Minhyeok Lee

**Affiliations:** 1 School of Electrical and Electronics Engineering, Chung-Ang University, Seoul 06974, Korea; 2 Department of Urology, Korea University College of Medicine, Seoul 02841, Korea

**Keywords:** asthma, air pollutants, linear correlation analysis, least absolute shrinkage and selection operator, random forest, hospital visits, national health insurance database

## Abstract

Asthma is a chronic respiratory disorder defined by airway inflammation, chest pains, wheezing, coughing, and difficulty breathing that affects an estimated 300 million individuals globally. Although various studies have shown an association between air pollution and asthma, few studies have used statistical and machine learning algorithms to investigate the effect of each individual air pollutant on asthma. The purpose of this research was to assess the association between air pollutants and the frequency of hospital visits by asthma patients using three analysis methods: linear correlation analyses were performed by Pearson correlation coefficients, and least absolute shrinkage and selection operator (LASSO) and random forest (RF) models were used for machine learning-based analyses to investigate the effect of air pollutants. This research studied asthma patients using the hospital visit database in Seoul, South Korea, collected between 2013 and 2017. The data set included outpatient hospital visits (n = 17,787,982), hospital admissions (n = 215,696), and emergency department visits (n = 85,482). The daily atmospheric environmental information from 2013 to 2017 at 25 locations in Seoul was evaluated. The three analysis models revealed that NO_2_ was the most significant pollutant on average in outpatient hospital visits by asthma patients. For example, NO_2_ had the greatest impact on outpatient hospital visits, resulting in a positive association (r=0.331). In hospital admissions of asthma patients, CO was the most significant pollutant on average. It was observed that CO exhibited the most positive association with hospital admissions (*I* = 3.329). Additionally, a significant time lag was found between both NO_2_ and CO and outpatient hospital visits and hospital admissions of asthma patients in the linear correlation analysis. In particular, NO_2_ and CO were shown to increase hospital admissions at lag 4 in the linear correlation analysis. This study provides evidence that PM_2.5_, PM_10_, NO_2_, CO, SO_2_, and O_3_ are associated with the frequency of hospital visits by asthma patients.

## 1. Introduction

Air pollution is described as the presence of substances in the air that are harmful to humans and linked to a higher risk of diseases [1,2,3,4,5], such as asthma, as a result of increased airway hyperresponsiveness. Various types of air pollutants, such as particulate matter (PM), nitrogen dioxide (NO_2_), carbon monoxide (CO), carbon dioxide (CO_2_), sulfur dioxide (SO_2_), and ozone (O_3_), have been found to trigger or exacerbate asthma attacks [6,7,8,9,10,11,12,13]. It is widely accepted that these air pollutants are emitted from various sources, including vehicles, manufacturing industries, and waste deposits. As the population of many urban centers in the developing world, such as in China and India, are expanding quickly, air pollution is worsening, and as a result, the risk of allergic and respiratory disease is increasing [14,15].

Asthma is a chronic respiratory disorder defined by airway inflammation, chest pains, wheezing, coughing, and difficulty breathing [16,17,18,19,20,21,22]. Asthma affects an estimated 300 million individuals globally, and the number continues to increase [23,24]. It has been discovered that solutions in improving air condition and relieving asthma are recommended, such as air filters, duct-type electrostatic precipitators (ESPs), and low-cost interventions (heating, ventilation, and air-conditioning; HVAC) [25,26,27]. These diverse approaches for improving air condition can be helpful; however, they should be preceded by the impact of air pollutants being determined in order to identify the underlying cause and seek appropriate solutions.

It has been widely observed that air pollution and the development of asthma are linked [28,29]. Although various studies have shown an association between air pollution and asthma, few studies have used statistical and machine learning algorithms to investigate the effect of each individual air pollutant on asthma. This study assessed the association between air pollutants and the frequency of hospital visits by asthma patients using three different analysis methods. Linear correlation analyses were performed by Pearson correlation coefficients, and least absolute shrinkage and selection operator (LASSO) and random forest (RF) models were used for machine learning-based analyses to investigate the effect of air pollutants.

While various studies have shown that air pollution aggravates asthma, the effects of the time lag between the air components and asthma, on the other hand, has gained less attention. The objective of this research was to examine the association between air pollutants and asthma, as well as to analyze the time lag effect using machine learning-based analyses, which can lead to more precise analyses. This research assessed daily atmospheric conditions using data from approximately 18 million hospital visits between 2013 and 2017 for asthma patients in Seoul, South Korea.

## 2. Materials and Methods

### 2.1. Seoul Citizen Hospital Visit Database for Asthma Patients

Seoul is the capital city of South Korea, with a population of over 10 million. This research studied asthma patients using the hospital visit database in Seoul. National health insurance is mandatory for all nationals in South Korea. Consequently, the National Health Insurance Service (NHIS) of South Korea has obtained all medical records of South Korean citizens. The NHIS has developed a hospital visit database for asthma patients that contains daily outpatient hospital visits, hospital admissions, and emergency department visits.

**Figure 1:**
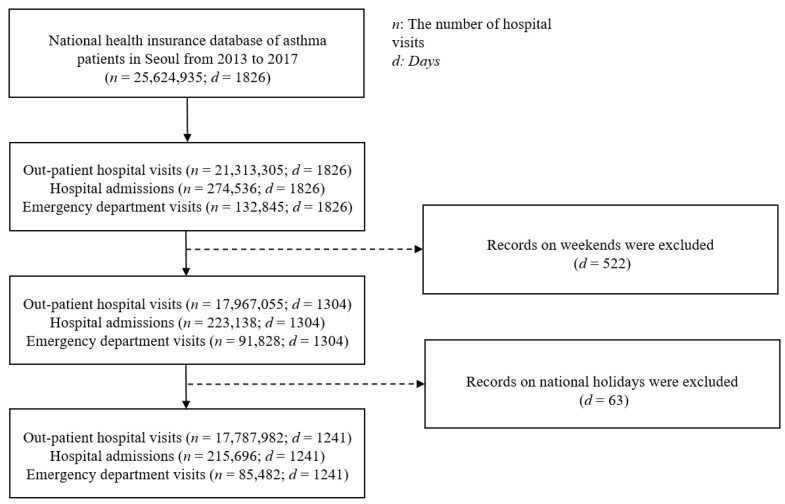
Preprocessing procedures for the database used in this study.

According to the database, asthmatic patients visited a hospital almost 26 million times between 2013 and 2017 in Seoul. Weekend records were excluded from this study since they indicated a different trend than weekdays due to hospital closures. For the same reason, records for 63 South Korean national holidays from 2013 to 2017 were removed. Thus, 17,787,982 outpatient hospital visits, 215,696 hospital admissions, and 85,482 emergency department visits for 1241 days were included. The data distributions for the hospital visits were log-normalized to transform them into Gaussian distributions for statistical analysis. An overview of the database preparation process is shown in Figure 1.

### 2.2. Daily Atmospheric Environmental Information Database

This study used a database containing daily atmospheric environmental information from 2013 to 2017 at 25 locations in Seoul. The data included averages of daily air pollutants at each location, including PM_10_, PM_2.5_, O_3_, NO_2_, CO, and SO_2_. Weekends and national holidays were excluded from the data in compliance with the hospital visit database.

### 2.3. Statistical and Machine Learning-Based Analyses

Linear correlation analyses were performed by Pearson correlation coefficients. For machine learning-based analyses, LASSO and RF were employed to investigate the effects of air pollutants and time lag associated with hospital visits by asthma patients.

LASSO is a statistical method that results in sparse models with few coefficients when used to regularize data models and feature selection [30]. The LASSO algorithm creates a penalty function to make some regression coefficient values closer to zero. This indicates that the less contributing variable is removed, and the more important contributing variable containing substantial data is selected to improve the interpretability of the model. Consequently, LASSO functions similarly to a structure selection method. The equation for LASSO is given as:

(1) $$\underset{\beta }{\mathrm{argmin}}\left\{\overset{n}{\underset{i=1}{\sum }}{\left(y_{i}-\underset{j}{\sum }x_{ij}\beta_{j}\right)}^{2}\right\}\;subject\;to\;\overset{p}{\underset{j=1}{\sum }}\left|\beta_{j}\right|\;\leq s,$$

where β denotes the coefficient vector and s denotes the predetermined free parameter that controls the degree of regularization. When the absolute value of the magnitude of β is large, it indicates that the *j-th* variable is a contributing variable. On the contrary, given a large enough penalty parameter s, the estimates of the absolute value of β shrink toward zero, suggesting that the variable is contributing less.

RF is a tree-based model that includes iteratively splitting a dataset into two groups based on a criterion until a stopping point is achieved. RF is employed by constructing multiple decision trees for both classification tasks and regression tasks [31]. Accordingly, RF can be a nonlinear approach to analyzing relationships between variables [32]. In the RF model, we can obtain variable importance that describes the effects of each variable for feature selection. The importance of the i-*th* feature is calculated as:

(2) $$I\left(f_{i}\right)=\frac{\sum w_{j}\cdot G\left(C_{j}\right)–w_{j_{left}}\cdot G\left(C_{j_{left}}\right)–w_{j_{right}}\cdot G\left(C_{j_{right}}\right)}{\sum I\left(C_{k}\right)},$$

where $C_{j}$ denotes the importance of node $C_{j}$ and w denotes the weight of node $C_{j}$, i.e., the ratio of the number of samples corresponding to node $C_{j}$ to the total number of samples, and $C_{k}$ denotes the sum of the importance of the nodes. Accordingly, $I\left(f_{i}\right)$ is defined as the sum of the importance of the nodes divided by the i-*th* feature compared to the sum of the importance of the entire nodes. Consequently, $I\left(f_{i}\right)$ in RF corresponds to the average of all $I\left(f_{i}\right)$ in each tree.

## 3. Results

### 3.1. Analysis of Hospital Visits and Air Pollutants Using Linear Correlation Analysis

The most correlated pollutant on average in outpatient hospital visits by asthma patients was NO_2_, which had a positive association (r=0.331), where *r* denotes the average correlation coefficient. The pollutant with the second-greatest impact on the frequency of outpatient hospital visits by asthma patients on average was CO by a 1.5 % difference (r=0.326).

**Figure 2:**
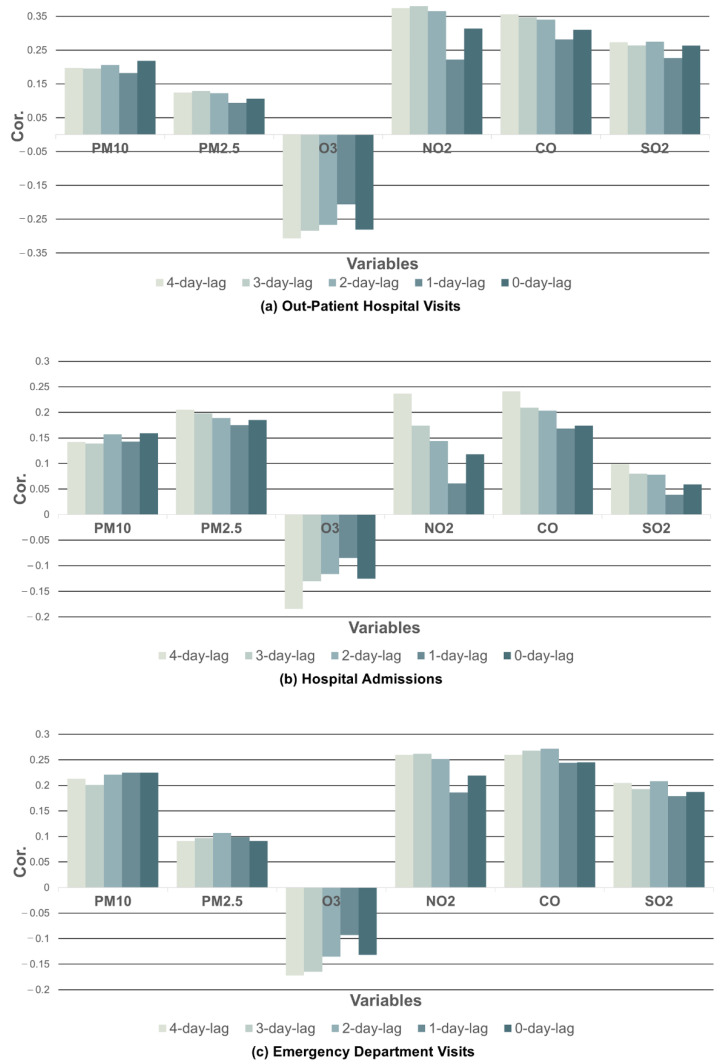
Coefficients between hospital visits and air pollutants using linear correlation analysis.

Separately, the association between each air pollutant level on the same day as the visits and 1 to 4 days prior to the visits was analyzed to examine the time lag effects. The NO_2_ level 3 days prior to the outpatient hospital visits had the most significant impact on the frequency of visits by asthma patients (r=0.380), where *r* denotes the correlation coefficient, which meant the NO_2_ level had the greatest effect on a visit in 3 days. Specifically, the association was followed by the NO_2_ level at 4 days *(*r=0.374) and 2 days *(*r=0.365) prior to outpatient hospital visits by asthma patients. The distributions of the correlation coefficients between each pollutant and outpatient hospital visits are shown in Figure 2a and Table 1.

In terms of hospital admissions of asthma patients, the most positively correlated pollutant on average was CO (r=0.199), followed by PM_2.5_ by a 4.5% difference (r=0.190).

For the time lag effect analysis, the CO level 4 days prior to the hospital admissions had the most significant effect on admission frequency (r=0.241) and the NO_2_ level 4 days prior to the visits had the second-greatest effect by a 1.6% difference (r=0.237). The distributions of the correlation coefficients between each pollutant and hospital admissions are shown in Figure 2b and Table 1.

On average, CO was the most correlated pollutant with emergency department visits by asthma patients (r=0.257), which resulted in a positive correlation. Accordingly, the association was followed by NO_2_ with an 8.5% difference compared to CO (r=0.235).

The CO level 2 (r=0.272) and 3 (*r* = 0.268) days prior to the emergency department visits had the most significant effects. Consequently, the NO_2_ level 3 days prior to the emergency department visits (r=0.262) followed in significance. The distributions of the correlation coefficients between each pollutant and emergency department visits by asthma patients are shown in Figure 2c and Table 1.

### 3.2. Analysis of Hospital Visits by Air Pollutants Using LASSO

The number of outpatient visits was positively correlated with NO_2_ (r=0.034) and PM_10_ (r=0.009) on average, where *r* denotes the averaged coefficient in LASSO. Additionally, it was found that PM_2.5_, O_3_, CO, and SO_2_ had a negative relationship with outpatient visits on average.

**Figure 3:**
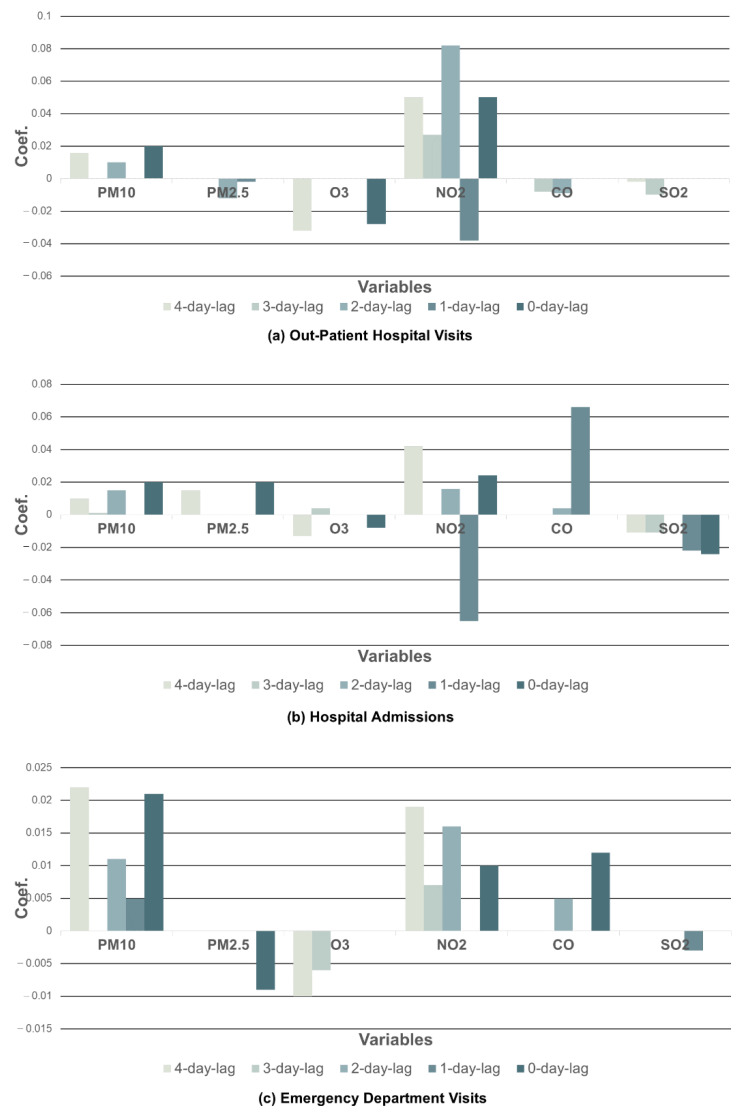
Coefficients between hospital visits and air pollutants using least absolute shrinkage and selection operator (LASSO).

For time lag effect analysis, the NO_2_ level 2 days prior to the visits (r=0.282) had the most significant impact on outpatient hospital visits by asthma patients, where *r* denotes the coefficient in LASSO. The distributions of LASSO coefficients between each pollutant and outpatient hospital visits are shown in Figure 3a and Table 2.

CO had the most significant effects on hospital admissions of asthma patients on average (r=0.014), resulting in a positive correlation. Consequently, the association was followed by PM_10_, with a 35% difference (r=0.009). It was observed that O_3_ and SO_2_ had negative correlations with hospital admissions on average. Separately, the CO level 1 day prior to the hospital admissions had the greatest impact, with significance (r=0.066), followed by the NO_2_ level 4 days prior to the visits (r=0.042). Conversely, the NO_2_ level 1 day prior to the hospital admissions significantly reduced the number of admissions (r=−0.065). The distributions of LASSO coefficients between each pollutant and hospital admissions are shown in Figure 3b and Table 2.

On average, PM_10_ (r=0.011), NO_2_ (r=0.010), and CO (r=0.003) were positively linked with the number of emergency department visits. On the contrary, PM_2.5_ (r=−0.011) and O_3_ (r=−0.003) had a negative relationship with emergency department visits. It was found that PM_10_ had the most significant time lag effect on emergency department visits, and the level 4 days prior to the visits showed a highly positive correlation (r=0.022). The distributions of LASSO coefficients between each pollutant and emergency department visits are shown in Figure 3c and Table 2.

### 3.3. Analysis of Hospital Visits by Air Pollutants Using RF

The feature or variable importance with RF describes which variables are relevant. The variable importance for each pollutant was averaged. The averaged results showed the same pattern as the results of the linear correlation analysis by Pearson correlation coefficients. The most significantly related pollutant with outpatient hospital visits by asthma patients was NO_2_ (*I* = 6.055)_,_ resulting in a positive association, where *I* denotes the variable importance of RF. The pollutant with the second-greatest impact on the frequency of outpatient hospital visits by asthma patients was CO (*I* = 4.229).

**Figure 4:**
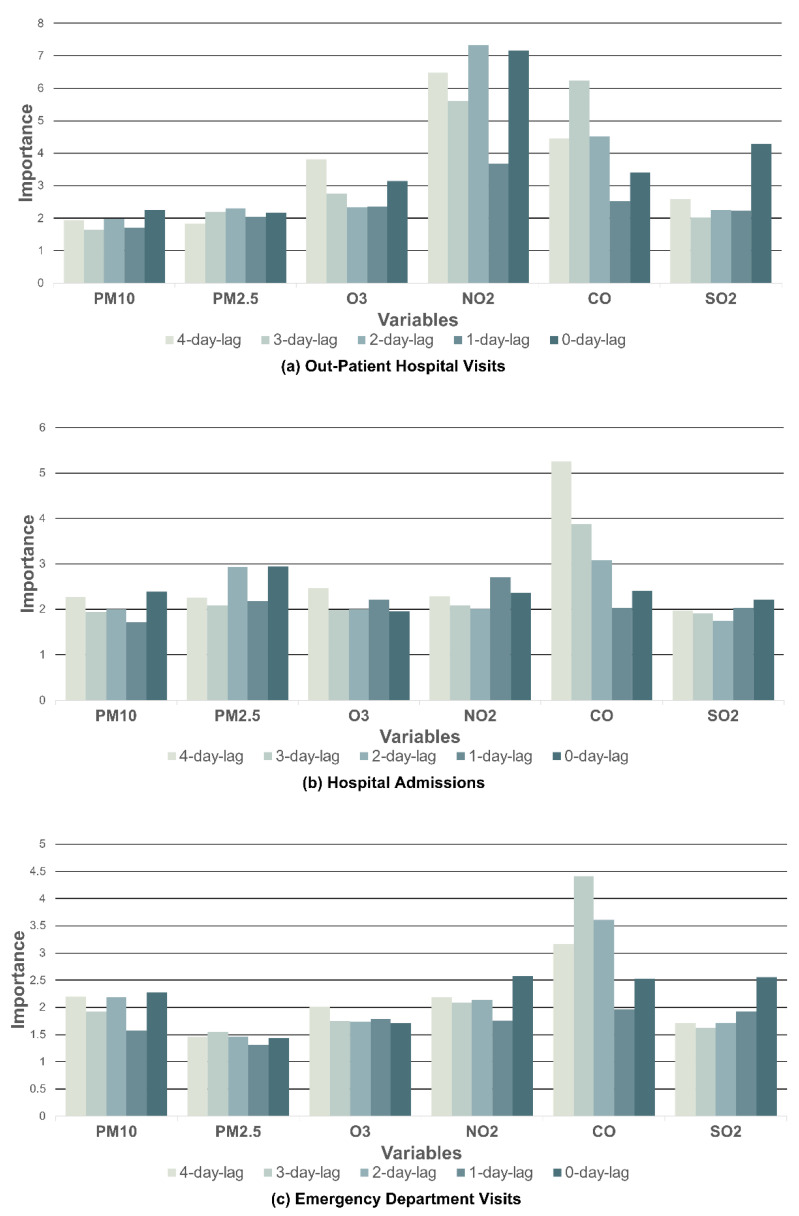
The variable importance of pollutants on hospital visits using the random forest (RF) model.

Individually, the NO_2_ level 2 days prior to the visits (*I* = 7.339) increased the frequency of the outpatient hospital visits considerably, which meant the NO_2_ level caused the greatest effects on a visit in 2 days. The distribution of variable importance of each pollutant for outpatient hospital visits is shown in Figure 4a and Table 3.

It was observed that CO exhibited the most positive association with hospital admissions on average (*I* = 3.329). This association was followed by PM_2.5_ with a 25% difference (*I* = 2.472). The distributions of variable importance of each pollutant for hospital admissions is shown in Figure 4b and Table 3.

CO had the greatest impact on emergency department visits by asthma patients, which resulted in a positive connection (*I* = 3.135). Consequently, NO_2_ had the second-greatest effect on the average number of visits by a 31% difference (*I* = 2.150). The distributions of variable importance of each pollutant for emergency department visits are shown in Figure 4c and Table 3.

For the individual time lag effect analysis, the CO level 4 days prior to the visits had a significant impact on the frequency of hospital admissions (*I* = 5.252). Specifically, the CO level 3 days before the emergency department visits significantly influenced the frequency of visits (*I* = 4.41).

## 4. Discussion

This study assessed the association between air pollutants and the frequency of hospital visits by asthma patients using three analysis methods. A relationship between air pollution and asthma has been proven in several studies [28,29,33,34], but there have been few studies that demonstrated the effect of multiple pollutants using statistical analyses and machine learning algorithms, whereas existing studies have focused on a single pollutant with conventional statistical analyses. However, in this study, various methods were employed, such as linear correlation analysis, LASSO, and RF, to establish that high levels of air pollutants result in an increase in hospital visits by asthma patients.

Numerous studies have shown that air pollution exacerbates asthma. The mechanisms that cause asthma exacerbation differ slightly depending on the kind of pollutants, but in general, alterations in lung function after exposure to air pollution are linked to an increased inflammatory response in the airways. Additionally, one study demonstrated that air pollution significantly enhances the likelihood of sensitization and reactions to inhaled allergens in asthma patients [19]. Another study demonstrated that asthma patients were sensitive even to low levels of SO_2_. When asthma patients exercise, bronchoconstriction occurs within a few minutes, even at low SO_2_ levels of 0.25 ppm. Also, it has been claimed that hospital admission for asthma is associated with PM_10_ levels [35]. Additionally, another study showed that an increase in NO_2_ and PM_10_ had a significantly positive effect on outpatient and emergency visits by asthma patients. The elevation of the NO_2_ level had the greatest impact on outpatient visits by asthma patients among pollutants, such as PM_10_, O_3_, NO_2_, and SO_2_ [36].

This study shows that the most significant air pollutant for outpatient hospital visits and hospital admissions was identical in the linear correlation, LASSO, and RF: it was demonstrated that NO_2_ had the most important impact on outpatient hospital visits and CO was the most significant for hospital admissions. For emergency department visits, linear correlation and RF showed the same trend. The results exhibit that CO and NO_2_ have a significant impact on emergency department visits using linear correlation and RF. On the contrary, PM_10_ was the most significant pollutant, which was followed by NO_2_ for emergency department visits using LASSO. The result signifies that NO_2_, which was a relatively more important feature than CO, was selected and the importance of CO was ignored. The reason for the difference between models was because each feature was analyzed independently in the linear correlation; however, each feature was relatively and comprehensively examined in LASSO. Accordingly, the result implied that NO_2_ and CO showed a significant correlation with each other; therefore, this raised the possibility that either NO_2_ or CO alone was required to evaluate the effect of the air pollutants since they indicated duplicated information.

In the linear correlation analysis of this study, it was demonstrated that NO_2_ and CO had a significant impact on outpatient hospital visits by asthma patients. Additionally, CO and PM_2.5_ significantly influenced hospital admissions of asthma patients. Contrarily, there was little evidence that the levels of O_3_ affected asthma. LASSO is a statistical method that results in sparse models with few coefficients when used to regularize data models and feature selection. The LASSO penalty pushes the coefficient value closer to zero if the corresponding variable has no influence on the target, or if the information in the variable is duplicated with other variables. It indicates that the less contributing variable is eliminated, and the more important contributing variable containing substantial data is selected. The LASSO model of this study assessed that NO_2_ had the most significant effect on outpatient hospital visits and CO significantly affected hospital admissions, which was the same trend as shown in the linear correlation analysis. For emergency department visits, PM_10_ had the most significant effect, distinct from the other two models. The RF model is a nonlinear approach to analyze relationships between variables. The variable importance in this model describes the effects of the variables. The average results of each hospital visit in the RF model were consistent with the linear correlation analysis. NO_2_ was found to have a significant effect on outpatient hospital visits for asthma patients, and CO was shown to have a significant effect on hospital admissions and emergency department visits. These same results from different models can further highlight the effects of each pollutant on asthma.

According to various studies, there is a time lag between air pollution and its effects, and the initiation of inflammatory responses and symptoms as a result of air pollutant exposure might occur during this time lag [37,38,39,40]. A study demonstrated that an increase in the PM_10_ level that occurred 2 days prior to the emergency department visits had a significant impact on the frequency of the visits [36]. These studies prompted us to investigate the link between delayed hospital visits and air pollution exposure. We used lags of 0–4 days for each pollutant to estimate the time lag effects. Our study found that there was a significant time lag between both NO_2_ and CO, and outpatient hospital visits and hospital admissions of asthma patients in the linear correlation analysis. In particular, NO_2_ and CO increased hospital admissions at lag 4 in the linear correlation analysis. As shown in the results of this study, there was a highly significant association between outpatient hospital visits for asthma and 2-day lags of NO_2_ and between hospital admissions for asthma and 1-day lags of CO in the LASSO model. Our study found that there was no remarkable time lag for emergency department visits in the linear correlation analysis and LASSO models, but 3-day lags of CO significantly influenced emergency department visits in the RF model. Also, there was a significant association with respect to the time lag effect between CO, outpatient hospital visits, and hospital admissions.

The findings of this study have a few limitations. Due to the limitations of the database, no subgroup analyses of asthma patients based on age, sex, or asthma severity were conducted. Other atmospheric characteristics, such as temperature, humidity, and weather, were not considered. There is a likelihood that the frequency of hospital visits is not proportional to the severity of the condition. Regardless of the limitations of these investigations, the three different analysis methods used, a large sample, and a long search can mitigate these limitations; hence, the conclusions of this study have been demonstrated by statistical and machine learning-based analyses. In the analyses of outpatient visits, there is a possibility that the number of visits on Mondays may be excessively measured due to the weekend closure of hospitals. This limitation might affect the results of the analyses in this study. However, it can be interpreted that effects and distortions from this limitation can be partially canceled out by the extensive study period, which covered 261 weeks. Additionally, since it is expected that the average numbers of visits on weekdays are similar, the number of visits on Monday can be preprocessed before the analyses in order to have the same average as the other weekdays by dividing the ratio between Monday and other weekdays, which can be studied further in future work.

## 5. Conclusions

This study provides evidence that PM_10_, PM_2.5_, NO_2_, CO, CO_2,_ SO_2,_ and O_3_ are associated with the frequency of hospital visits by asthma patients. Furthermore, our research suggests that air pollution may have a priming impact on the inflammatory response, causing the lungs to become more susceptible to inflammatory stimuli, assuming that there is a time lag effect between some pollutants and asthmatic hospital admissions.

## Figures and Tables

**Table 1:** Coefficients between hospital visits and air pollutants using linear correlation analysis. The intervals represent 95% confidence intervals.

		PM_10_	PM_2.5_	O_3_	NO_2_	CO	SO_2_
Outpatient Hospital Visits	4-day lag	0.197 ±0.0004	0.124 ±0.0005	−0.307 ±0.0004	0.374 ±0.0004	0.356 ±0.0004	0.273 ±0.0004
	3-day lag	0.195 ±0.0004	0.129 ±0.0005	−0.284 ±0.0004	0.380 ±0.0004	0.347 ±0.0004	0.264 ±0.0004
	2-day lag	0.206 ±0.0004	0.123 ±0.0005	−0.267 ±0.0004	0.365 ±0.0004	0.340 ±0.0004	0.275 ±0.0004
	1-day lag	0.182 ±0.0004	0.094 ±0.00046	−0.206 ±0.0004	0.222 ±0.0004	0.281 ±0.0004	0.226 ±0.0004
	0-day lag	0.218 ±0.0004	0.106 ±0.0005	−0.281 ±0.0004	0.314 ±0.0004	0.310 ±0.0004	0.263 ±0.0004
Hospital Admissions	4-day lag	0.142 ±0.0041	0.205 ±0.0040	−0.184 ±0.0042	0.237 ±0.0040	0.241 ±0.0040	0.099 ±0.0042
	3-day lag	0.139 ±0.0041	0.198 ±0.0041	−0.13 ±0.0042	0.174 ±0.0041	0.209 ±0.0040	0.08 ±0.00419
	2-day lag	0.157 ±0.0041	0.189 ±0.0041	−0.116 ±0.0042	0.144 ±0.0041	0.203 ±0.0040	0.078 ±0.0042
	1-day lag	0.143 ±0.0041	0.175 ±0.0041	−0.085 ±0.00419	0.061 ±0.0041	0.168 ±0.0041	0.039 ±0.0042
	0-day lag	0.159 ±0.0041	0.185 ±0.0041	−0.125 ±0.0042	0.118 ±0.0042	0.174 ±0.0041	0.059 ±0.0042
Emergency Department Visits	4-day lag	0.213 ±0.0064	0.091 ±0.0064	−0.172 ±0.0065	0.260 ±0.0063	0.260 ±0.0062	0.205 ±0.0064
	3-day lag	0.201 ±0.0064	0.097 ±0.0066	−0.165 ±0.0065	0.262 ±0.0062	0.268 ±0.0062	0.193 ±0.0064
	2-day lag	0.221 ±0.0064	0.107 ±0.0066	−0.135 ±0.0066	0.252 ±0.0063	0.272 ±0.0062	0.208 ±0.0064
	1-day lag	0.225 ±0.0064	0.099 ±0.0066	−0.093 ±0.0066	0.186 ±0.0065	0.244 ±0.0063	0.179 ±0.0065
	0-day lag	0.225 ±0.0064	0.091 ±0.0064	−0.132 ±0.0066	0.219 ±0.0064	0.245 ±0.0063	0.187 ±0.0065

**Table 2:** Coefficients between hospital visits and air pollutants using least absolute shrinkage and selection operator (LASSO).

		PM_10_	PM_2.5_	O_3_	NO_2_	CO	SO_2_
Outpatient Hospital Visits	4-day lag	0.016	0	−0.032	0.05	0	−0.002
	3-day lag	0	0	0	0.027	−0.008	−0.010
	2-day lag	0.010	−0.012	0	0.082	−0.009	0
	1-day lag	0	−0.002	0	−0.038	0	0
	0-day lag	0.020	0	−0.028	0.050	0	0
Hospital Admissions	4-day lag	0.010	0.015	−0.013	0.042	0	−0.011
	3-day lag	0.001	0	0.004	0	0	−0.011
	2-day lag	0.015	0	0	0.016	0.004	0
	1-day lag	0	0	0	−0.065	0.066	−0.022
	0-day lag	0.020	0.020	−0.008	0.024	0	−0.024
Emergency Department Visits	4-day lag	0.022	0	−0.010	0.019	0	0
	3-day lag	0	0	−0.006	0.007	0	0
	2-day lag	0.011	0	0	0.016	0.005	0
	1-day lag	0.005	0	0	0	0	−0.003
	0-day lag	0.021	−0.009	0	0.010	0.012	0

**Table 3:** The variable importance of pollutants on hospital visits using the random forest (RF) model.

		PM_10_	PM_2.5_	O_3_	NO_2_	CO	SO_2_
Outpatient Hospital Visits	4-day lag	1.939	1.839	3.820	6.478	4.454	2.594
	3-day lag	1.647	2.195	2.767	5.616	6.230	2.014
	2-day lag	1.981	2.302	2.333	7.339	4.518	2.250
	1-day lag	1.711	2.041	2.361	3.682	2.531	2.229
	0-day lag	2.251	2.180	3.151	7.160	3.412	4.284
Hospital Admissions	4-day lag	2.274	2.249	2.467	2.279	5.252	1.967
	3-day lag	1.940	2.085	1.984	2.083	3.883	1.915
	2-day lag	2.003	2.931	2.002	2.010	3.082	1.742
	1-day lag	1.719	2.176	2.214	2.705	2.023	2.035
	0-day lag	2.386	2.944	1.952	2.365	2.408	2.217
Emergency Department Visits	4-day lag	2.199	1.461	2.017	2.188	3.164	1.710
	3-day lag	1.925	1.552	1.745	2.088	4.410	1.624
	2-day lag	2.192	1.466	1.742	2.144	3.607	1.714
	1-day lag	1.577	1.319	1.786	1.753	1.972	1.928
	0-day lag	2.275	1.441	1.710	2.577	2.526	2.555

## Data Availability

The national health insurance data and atmospheric environmental data used in this study are public data available at https://nhiss.nhis.or.kr/bd/ab/bdabf001cv.do (accessed on 8 April 2022) and https://data.seoul.go.kr/dataList/OA-2220/S/1/datasetView.do (accessed on 8 April 2022), respectively.
